# Application of Lanthanum at the Heading Stage Effectively Suppresses Cadmium Accumulation in Wheat Grains by Downregulating the Expression of *TaZIP7* to Increase Cadmium Retention in Nodes

**DOI:** 10.3390/plants13202921

**Published:** 2024-10-18

**Authors:** Caixia Xiao, Hua Yang, Xingwang Chen, Jie Li, Xiongfei Cai, Jian Long

**Affiliations:** 1 School of Geography and Environmental Sciences, Guizhou Normal University, Guiyang 550025, China; siyue2127@163.com (C.X.); cxingwang2024@163.com (X.C.);; 2 The State Key Laboratory Incubation Base for Karst Mountain Ecology Environment of Guizhou Province, Guiyang 550001, China; 3 Guizhou Key Laboratory of Mountain Environment, Guizhou Normal University, Guiyang 550001, China; longjian22@163.com

**Keywords:** lanthanum, cadmium, wheat, nodes, accumulation, *TaZIP7*

## Abstract

Reducing cadmium (Cd) accumulation in wheat is an effective way to decrease the potential threats of Cd to human health. The application of lanthanum (La) in agricultural fields is eliciting extensive attention due to its beneficial effects on improving yields and inhibiting Cd accumulation in edible parts of crops. However, the potential mechanism of La-restricted Cd accumulation in crop grains is not entirely understood. Here, we investigated the effects of La and Cd accumulation in wheat grains by implementing application at the shooting and heading stages. Some associated mechanisms were explored. Results showed that La application at the shooting and heading stages considerably promoted the thousand-grain weight. La application at the shooting and heading stages increased Cd accumulation in the first node beneath the panicle (N1) but reduced Cd levels in the other tissues. La application at the heading stage exerted greater effects on Cd storage in N1 while reducing Cd concentrations in the other tissues compared with La application at the shooting stage. La addition substantially decreased the translocation of Cd from the lower nodes to the upper internodes, but increased Cd translocation from the lower internodes to the upper nodes. The expression of *TaZIP7* in N1 was downregulated by La treatment. These results suggest that the effective reduction in Cd in wheat grains by La application at the heading stage is probably a consequence of the successful promotion of Cd storage in nodes by downregulating the expression of *TaZIP7* during the grain-filling stage, thereby hindering the redirection Cd from nodes to grains.

## 1. Introduction

Cadmium (Cd) is a toxic element classified as a Class 1 carcinogenic substance [1]. Excess Cd can remain in the skeletal system, liver, kidney, and reproductive organs, and its accumulation in these organs has a biological half-life of 10–33 years [2]. Cd can also lead to other related severe ailments, such as osteoporosis, renal dysfunction, and hypertension [3,4]. According to the China Ecological Environment Bulletin, Cd is one of the most prevalent contaminants that affect the quality of agricultural lands [5]. Cd can be easily absorbed and accumulated in the edible parts of crops, thus endangering public health through the food chain [6]. Wheat is one of the most vital food crops globally. In 2022, approximately 138 million tons of wheat, which account for 20.1% of all grain crops, were produced in China [7]. The grain Cd content of wheat and rice in several regions is higher than the maximum permissible threshold of 0.2 mg/kg recommended by FAO/WHO, which leads to health risks with long-term consumption of Cd-contaminated cereals [8]. Wheat has elicited increasing concern because it can accumulate large amounts of Cd by dominantly translocating Cd from roots to shoots and eventually accumulating it in grains [9,10,11]. Therefore, Cd levels in wheat grains need to be reduced.

Rare earth elements (REEs) are composed of scandium, yttrium, and 15 kinds of lanthanide elements in the periodic table of elements with uniform chemical properties [12]. REEs possess a distinctive characteristic; that is, they can attach themselves to other molecules [13,14,15,16]. Compared with other REEs, lanthanum (La) exists in larger quantity in the environment and serves as the primary constituent of commercially available rare earth microfertilizers [17]. Research has shown that a low dose of La helps to promote the germination of rice seeds [18], the growth of root organogenesis [19,20], 2-acetyl-1-pyrroline biosynthesis [21], photosynthetic characteristics, and antioxidant activity [16]. Several studies have also confirmed that La can successfully mitigate Cd toxicity and inhibit Cd accumulation in crops [22,23,24].

Cd is loaded into the grains of a crop via four pathways, namely, root uptake from the soil, root-to-shoot translocation by xylem loading, redirection in nodes by vascular bundles, and remobilization from leaves to grains via the phloem [25]. *Nramp5* is involved in the absorption of Cd from the soil solution to the root exodermis and endodermis and subsequently to pericycle and parenchyma cells in the steles via the symplastic pathway [26]. Knockout of *OsNramp5* is regarded as an effective approach to minimize Cd uptake and accumulation in rice [27]. *OsHMA3* has been reported to govern the vacuole sequestration of Cd in root cells of rice [9]. Wiggenhauser et al. [28] found that downregulation of *OsHMA3* leads to increased Cd translocation from rice root to shoot. After root uptake, Cd is loaded into the xylem by *HMA2*, which is a major efflux transporter of Zn localized to the plasma membrane of the pericycle cells of roots [29]. Overexpression of *OsHMA2* favors increased Cd concentration in the shoots of rice during the vegetative stage [30]. After xylem loading, Cd is preferentially transferred to the upper nodes and further into the panicle [4]. Nodes are major means of redirecting Cd via intervascular transfer, which can effectively limit and reallocate Cd in other tissues during the grain-filling stage [25,31,32]. *OsZIP7* and *OsHMA2* in nodes facilitate the intervascular transfer of Zn and Cd from nodes to rice grains [33]. *OsZIP7* is strongly expressed in the parenchyma cells of vascular bundles in nodes. Knockout of *OsZIP7* results in increased retention of Cd and Zn in the basal node region [33]. *OsHMA2* is highly expressed at the phloem of enlarged vascular bundles (EVBs) and diffuse vascular bundles (DVBs) in nodes [25]. Moreover, Rodda et al. [34] reported that approximately 60% of the final Cd in grain is remobilized from the Cd accumulated by the plant prior to flowering at maturity. *OsLCT1* is a Cd-efflux transporter localized at the plasma membrane in nodes and involved in the reactivation of Cd from leaves via the phloem [35]. Knockdown of *OsLCT1* has been found to lead to decreased Cd accumulation in rice grains [36]. Yang et al. [23] discovered that reduced Cd accumulation in wheat grains by La supply is due to decreased Cd absorption and root-to-shoot translocation through the downregulation of *TaNramp5* and *TaHMA2*. However, whether La can effectively manipulate *TaHMA2*, *TaZIP7*, and *TaLCT1* in nodes to restrict the redirection and remobilization of Cd is unknown.

A previous study reported that the grain-filling stage from anthesis to maturity is vital for the formation of healthy grain [37] and the key period for determining the final Cd level in grains [38]. Therefore, compared with the application of La at the shooting stage of wheat, La treatment at the heading stage could have a more remarkable effect on the reduction in Cd accumulation in wheat grains by regulating the expression of *TaHMA2*, *TaZIP7*, and *TaLCT1* in nodes to suppress the redirection and remobilization of Cd. In this study, pot experiments were conducted to (1) determine if La application at the heading stage of wheat has a more remarkable effect on the reduction in Cd accumulation in wheat grains than at the shooting stage and (2) find out if this remarkable effect on the reduction in Cd accumulation could be related to the restriction of Cd redirection and remobilization resulting from the regulated expression of *TaHMA2*, *TaZIP7*, and *TaLCT1* in nodes.

## 2. Materials and Methods

### 2.1. Experimental Design

The soil used in this experiment was collected from the plow layer (0–20 cm) of a paddy field located in Xiniu Village, Qingzhen City, Guizhou Province, China. The principal physico-chemical characteristics of the soil are presented in Table S1 (Supplementary Materials). Specifically, 0.6 mg·kg^−1^ Cd (CdCl_2_·2.5 H_2_O) was spiked to the soil after air drying and made to pass through a 2 mm sieve. The Cd-contaminated soil was placed in a plastic bag, and its field water holding capacity (60%) was maintained with tap water. After 30 days, basal fertilizers (100 mg·kg^−1^ N as urea, 100 mg·kg^−1^ P as KH_2_PO_4_, and 50 mg·kg^−1^ K as potassium sulfate) were mixed thoroughly into the soil. Then, the soil was placed in polyethylene pots (26.5 cm × 26.5 cm × 23.0 cm) with 7.5 kg of soil in each pot. In accordance with the method described by Yang, et al. [23], wheat seeds (*Triticum aestivum* L. cv. Yaomai 16) provided by Shanxi Academy of Agricultural Sciences were evenly sown in pots (six seeds per pot). The wheat seedlings were thinned to three plants per pot at the two-leaf stage. Three treatments were included in this study: (1) Cd (sole Cd with no La application marked as control); (2) 1 mg·kg^−1^ La was applied at the shooting stage under Cd stress; and (3) 1 mg·kg^−1^ La was applied at the heading stage under Cd stress. The pot experiment was conducted at Guizhou Normal University (Guiyang, China) with natural sunlight and ambient temperature from October 2021 to May 2022. 

**Figure 1:**
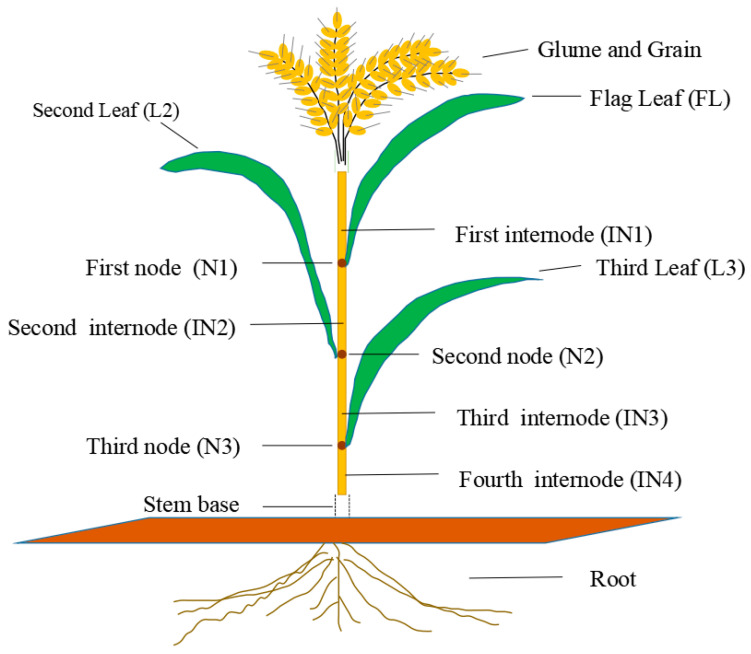
The image shows the location of each sampled section in a wheat plant.

The whole plants were harvested when the grains were ripe. The roots were rinsed with tap water, and the whole plants were washed with deionized water. The plant height of each plant was recorded. The wheat plants, including the grains, glumes, first internode (IN1), first node (N1), flag leaf (FL), second internode (IN2), second node (N2), second leaf (L2), third internode (IN3), third node (N3), third leaf (L3), fourth internode (IN4), and roots, were sampled separately from top to bottom (Figure 1). The samples were sterilized at 105 °C in an oven for 30 min and subsequently oven-dried at 75 °C to a relatively constant weight. Shoot dry weight, root dry weight, and thousand-grain weight were measured.

### 2.2. Relative Expression Analysis of Genes

At the heading stage, the relative expression of wheat genes, including TaHMA2, TaZIP7, and TaLCT1, in N1 that are potentially involved in the redirection and remobilization of Cd, was analyzed. N1 with or without 1 mg·kg^−1^ La at the heading stage were sampled and frozen in liquid nitrogen after 24 h. Total RNA was isolated using the Trizol reagent, and 1 μg of total RNA was employed for quantitative reverse transcription polymerase chain reaction (qRT-PCR). The reaction mixture consisted of 0.2 µL of forward primer, 0.2 µL of reverse primer, 1.0 µL of cDNA template, 3.6 µL of ddH_2_O, and 5 µL of SybrGreen qPCR Master Mix. The reaction was performed as 45 cycles of 3 min of initial steps at 95 °C, secondly for 15 s of melting at 95 °C, and a final annealing/extension at 60 °C for 30 s by using LightCycler 480 II fluorescence quantitative PCR machine (Sangon Biotech, Shanghai, China). The primer sequences used for qRT-PCR are presented in Table S2. OsHistoneH3 was adopted as an internal standard gene [39]. The relative expression of the target gene was calculated with the 2^−(∆∆Ct)^ method.

### 2.3. Element Analysis

To determine the total Cd and La concentrations, the plant samples were ground to a fine powder. Approximately 0.2 g of powder was acidified with a high-purity acid mixture of HNO_3_/HClO_4_ (4:1, *v*/*v*) in accordance with a previous study [22]. After the digest was properly diluted with 1% HNO_3_, the contents of Cd and La in the digestion solution were analyzed by inductively coupled plasma mass spectrometry (NexION 1000; PerkinElmer, Hopkinton, MA, USA). Quality assurance and quality control (QA/QC) were measured with duplicates, reagent blanks (acid mixture without sample), matrix spikes, and certified reference materials (CRMs) (citrus leaves, GBW10020(GSB–11)).

### 2.4. Statistical Analysis

Translocation factor (TF) is an index used to evaluate the transport ability of heavy metals from lower to upper tissues [31]. In accordance with the method reported by Liu et al. [40], TF was calculated as the ratio of the concentration of Cd in the upper tissues to that in the lower tissues of the plant (Formula (1)). The bioconcentration factor (BCF) was calculated as the ratio of the concentration of Cd in the wheat roots to that in the soil solution [41] (Formula (2)) as follows:

(1) $${TF}_{a-b}=\frac{C_{b}}{C_{a}},$$

(2) $$BCF=\frac{C_{Root}}{C_{Soil}},$$

where C*a* and C*b* are the Cd concentrations in the lower and upper tissues, respectively.

In accordance with the method described by Zhou et al. [31], the weighted average concentrations of Cd in the stem (Formula (3)) and leaf (Formula 4) were calculated based on the concentrations of dry weight (DW), node (N), internode (IN), and leaf (L) as follows:

(3) $${Cd}_{stem}=\frac{\sum_{i=1}^{n}\left({Cd}_{Ni}\times {DW}_{Ni}+{Cd}_{INi}\times {DW}_{INi}\right)}{\sum_{i=1}^{n}\left({DW}_{Ni}+{DW}_{INi}\right)},$$

(4) $$Cd_{\mathrm{leaf}}=\frac{\sum_{i=1}^{n}\left({Cd}_{Li}\times {DW}_{Li}\right)}{\sum_{i=1}^{n}{DW}_{Li}},$$

where *i* represents 1, 2, 3, or 4; Cd_Ni_, Cd_INi_, and Cd_Li_ are the Cd concentrations in N_i_, IN_i_, and L_i_, respectively; and DW_Ni_, DW_INi_, and DW_Li_ are the dry weights of N_i_, IN_i_, and L_i_, respectively. The calculation method of the weighted average concentration of La in the stems and leaves is similar to that of Cd.

SPSS 26.0 software was employed for the statistical analysis of the data. All data were presented as mean ± standard deviation (SD, *n* = 3). Duncan’s test was used to analyze the significant differences in metal concentrations in different wheat parts, BCF, and TF among various treatments (*p* < 0.05). Origin 2023 was used to plot the figures.

## 3. Results

### 3.1. Effects of La Application at Different Growth Stages on the Growth Performance of Wheat Under Cd Stress

**Figure 2:**
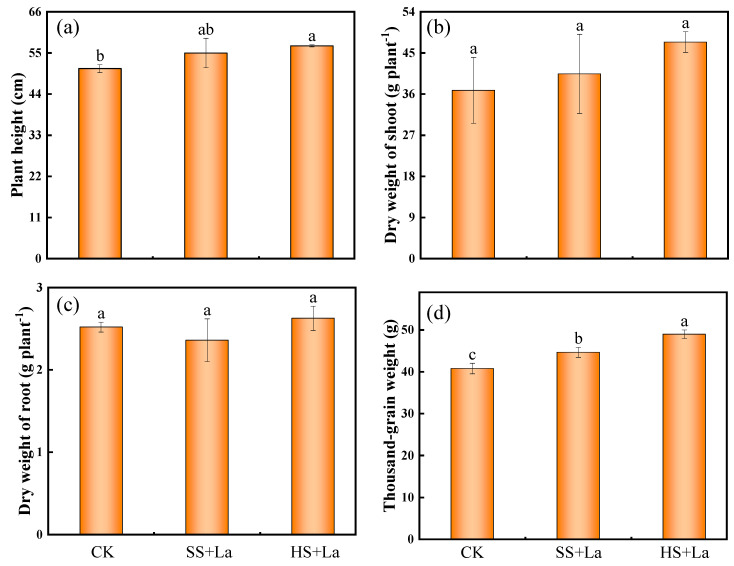
Effects of La application at different growth stages on agronomic traits of wheat. Plant height (**a**), dry weight of shoots (**b**), dry weight of roots (**c**), and thousand-grain weight (**d**). CK, SS + La and HS + La represent control, La supplementation at shooting stage and La supplementation at heading stage, respectively. Error bars represent standard deviation. Significant differences among treatments are indicated by different letters according to the Duncan’ test (*p* < 0.05).

The application of La at the different growth stages did not remarkably change the shoot dry weight (Figure 2b) and root dry weight (Figure 2c) compared with the application of Cd stress only. Plant height (Figure 2a) and thousand-grain weight (Figure 2d) were increased by La supplementation at the shooting and heading stages, respectively. Particularly, the thousand-grain weight (Figure 2d) amounted to 44.63 ± 0.12 g and 48.97 ± 0.10 g after La treatment at the shooting and heading stages, respectively; these values are significantly higher than the value of 40.76 ± 0.13 g without La addition (*p* < 0.05). The thousand-grain weight increased by 9.49% and 20.14%.

### 3.2. Effects of La Application at Different Growth Stages on Cd Accumulation in the Roots, Stems, Leaves, Glumes, and Grains of Wheat

**Figure 3:**
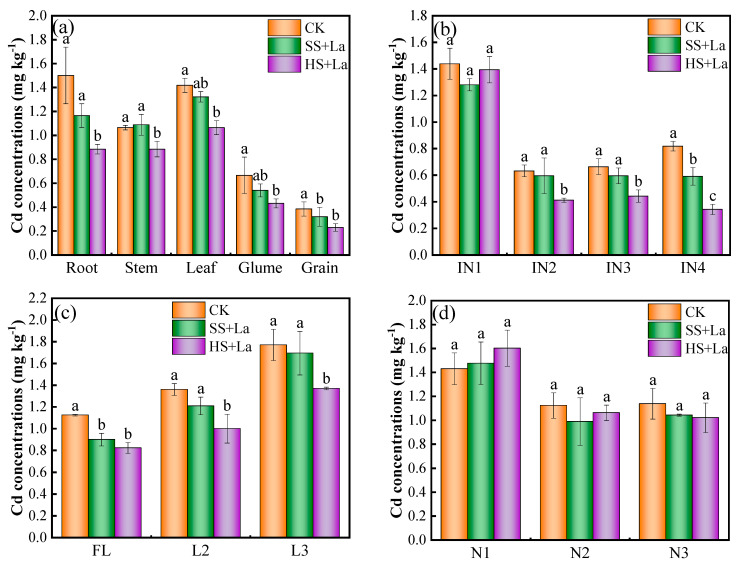
Cd concentrations in the roots, stems, leaves, glumes and grains (**a**), IN1, IN2, IN3 and IN4 (**b**), FL, L2, L3 (**c**), N1, N2 and N3 (**d**) of wheat treated with La at different growth stages. Where Ni represents the i-th node, INi represents the i-th internode, Li represents the i-th leaf and FL represents the first leaf (n = 3). Error bars represent standard deviation. Significant differences among treatments are indicated by different letters according to the Duncan’ test (*p* < 0.05).

The Cd concentrations in the roots, stems, leaves, glumes, and grains of wheat treated with La at different growth stages are presented in Figure 3a. The Cd concentrations in the roots, stems, leaves, glumes, and grains decreased remarkably regardless of the stages of La application, with the exception of the slight increase in the stems with La treatment at the shooting stage. Moreover, the application of La at the heading stage decreased the Cd concentrations in these tissues more effectively than La supplementation at the shooting stage did. La treatment at the heading stage remarkably reduced the Cd concentrations in the roots, stems, leaves, and glumes by 69.69%, 20.37%, 33.31%, and 54.32%, respectively, relative to the control. Particularly, La application at the heading stage significantly decreased the Cd concentration in the grains by 67.43% compared with the control (*p* < 0.05).

**Figure 4:**
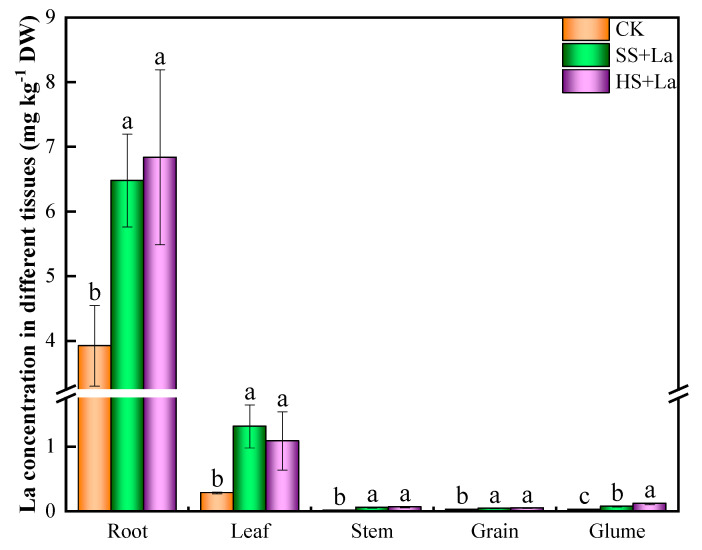
La Concentrations in roots, leaves, stems, grains and glumes of wheat treated with La at different growth stages. Error bars represent Standard deviation. Significant differences among treatments are indicated by different letters according to the Duncan’ Test.

The application of La considerably increased the La concentrations in wheat’s roots, stems, leaves, glumes, and grains, and nearly all of the La was located in the roots and leaves (Figure 4).

### 3.3. Effects of La Application at Different Growth Stages on Cd Accumulation in the Functional Internodes, Leaves, and Nodes of Wheat

The Cd concentrations differed in the functional internodes, leaves, and nodes at different locations in the control and La application treatments (Figure 3). From IN4 to IN1, the Cd concentrations were in the ranges of 0.34–0.82, 0.44–0.66, 0.41–0.63, and 1.35–1.44 mg·kg^−1^ for the control, La supplementation at the shooting stage, and La supplementation at the heading stage, respectively (Figure 3b). IN1’s Cd concentrations were approximately 1.75–2.27, 2.14–2.16, and 3.04–3.93 folds higher than those of IN2–IN4, respectively, under the same treatment. La application significantly inhibited Cd accumulation in IN2–IN4 at the heading stage in comparison with Cd stress alone (*p* < 0.05). From L3 to FL, the Cd concentrations were in the ranges of 1.07–1.77, 0.90–1.69, and 0.82–1.37 mg·kg^−1^ for the control, La application at the shooting stage, and La application at the heading stage, respectively. Among the three leaves, L3 had the highest Cd concentrations, which were about 1.30–1.65, 1.41–1.88, and 1.37–1.67 folds higher than those of L2 and FL under the same treatment (Figure 3c). La application at the heading stage of wheat remarkably decreased Cd accumulation in all the leaves. However, no statistically significant difference was observed in La supply at the shooting stage. The Cd concentrations in N1, N2, and N3 were comparable and had ranges of 1.43–1.60, 0.99–1.12, and 1.02–1.14 mg·kg^−1^ for the control, La treatments at the shooting stage, and La treatments at the heading stage, respectively (Figure 3d). Among the nodes, N1 had the highest Cd concentrations, which were approximately 1.25–1.28, 1.41–1.48, and 1.51–1.57 folds higher than those in N2 and N3 under the same treatment. Compared with the control, La treatment at the heading stage reduced the Cd accumulation in N2 and N3. The Cd concentrations in N2 and N3 decreased by 11.96% and 8.38% with La supply at the shooting stage and by 5.40% and 10.33% with La supply at the heading stage, respectively. La application at the shooting and heading stages increased Cd accumulation in N1, and the values became higher by 3.24% and 12.02% than those in the non-La-supplied plants.

### 3.4. Distribution of Cd in Different Tissues of Wheat with La Treatment at Different Growth Stages

**Figure 5:**
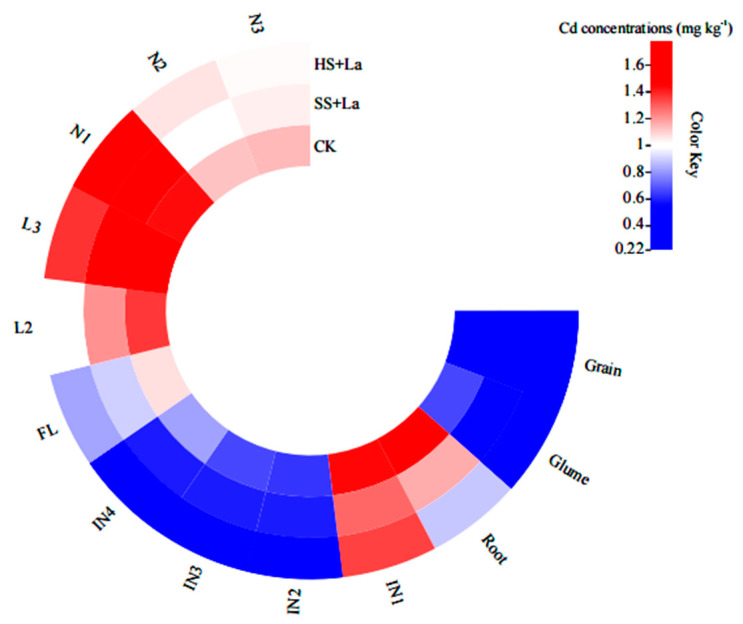
Heat map of the Cd concentrations in different tissues of wheat. The color depth represents the Cd concentration (*p* < 0.05).

**Figure 6:**
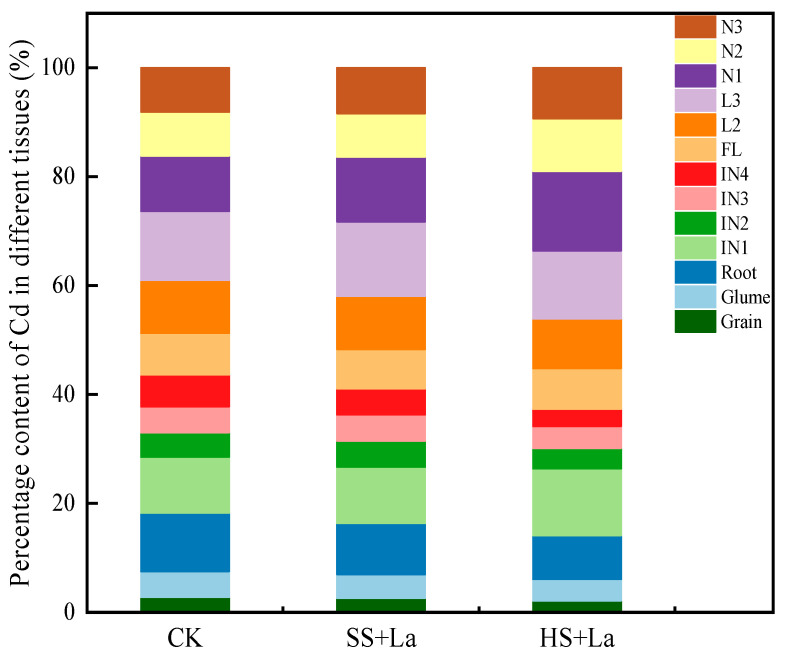
Distribution of Cd in wheat with La treatment at different growth stages.

A heat map was plotted to display the distribution of Cd in 13 tissues of wheat (Figure 5). Generally, La application at the shooting and heading stages reduced the Cd levels in most of the wheat tissues (except for N1), but La application at the heading stage had a stronger effect than La application at the shooting stage. The distribution proportion of Cd in the different tissues is further illustrated in Figure 6. After La was supplied at the shooting stage, the proportion of Cd in IN1, N1, IN2, L2, IN3, N3, and L3 increased by 0.53%, 16.58%, 6.28%, 0.32%, 1.25%, 3.46%, and 8.06%, respectively, and that in the grains, glumes, FL, N2, IN4, and roots decreased by 6.13%, 8.43%, 5.29%, 0.59%, 18.37%, and 12.38%, respectively. After La was supplied at the heading stage, the proportion of Cd in IN1, N1, N2, and N3 increased by 19.44%, 43.00%, 20.76%, and 14.48%, respectively, and that in the grains, glumes, FL, IN2, L2, IN3, L3, IN4, and roots decreased by 23.75%, 17.27%, 2.09%, 16.87%, 6.15%, 14.79%, 1.27%, 46.54%, and 24.77%, respectively.

### 3.5. Effects of La Application at Different Growth Stages on the Transport Characteristics of Cd

**Figure 7:**
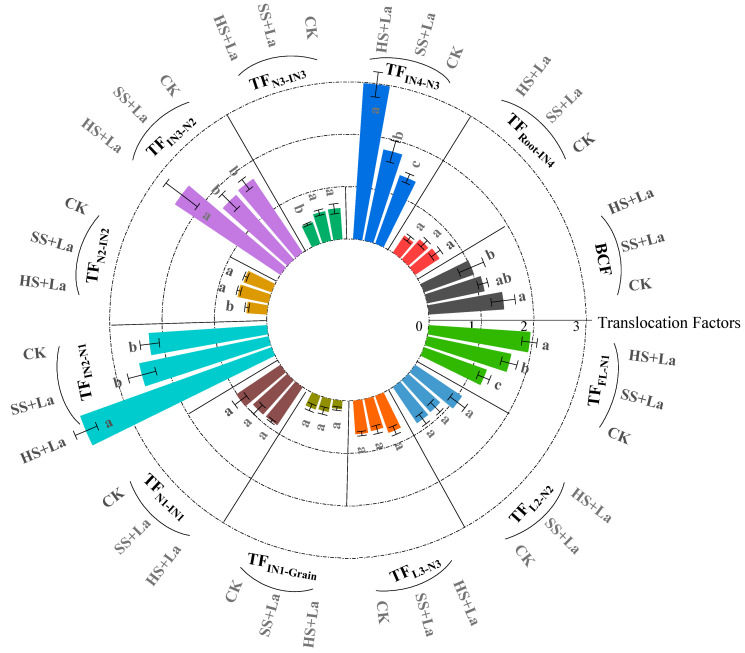
Effect of La application at different growth stages on the BCFS and TFs of Cd in wheat plants. Error bars represent standard deviation. Significant differences among treatments are indicated by different letters according to the Duncan’ test (*p* < 0.05).

The effects of La application at different growth stages on the transport characteristics of Cd are given in Figure 7. The TF_INi+1-Ni_ values of Cd were greater than 1 in all the treatments and gradually increased as the location rose. Notably, La application at different growth stages increased the TF_INi+1-Ni_ values of Cd. La supplementation at the heading stage had a stronger effect on the TF_INi+1-Ni_ values of Cd than La supplementation at the shooting stage. The TF_Ni-INi_ values of Cd were less than 1 in all the treatments. TF_N3-IN3_, TF_N1-IN1_, and TF_N2-IN2_ were reduced by La application at different growth stages. Similarly, La supplementation at the heading stage had a stronger influence on the TF_Ni-INi_ values of Cd than La supplementation at the shooting stage. The TF_Li-Ni_ values of Cd increased as the location rose in all the treatments. La supply promoted the TF_Li-Ni_ values of Cd, and La supplementation at the heading stage had a stronger influence on the TF_Li-Ni_ values of Cd than La supplementation at the shooting stage.

### 3.6. Effects of La Application at the Heading Stage on the Expression of Genes in N1 of Wheat

**Figure 8:**
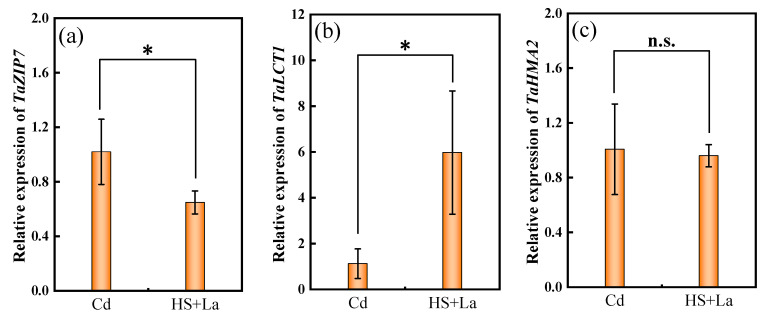
Effect of La application and lack thereof at the heading stage on expression of *TaZIP7* (**a**), *TaLCT1* (**b**), and *TaHMA2* (**c**) in N1 of wheat under Cd treatment. The data are expressed as mean ± standard deviation. * (*p* < 0.05) and n.s. indicate significant or insignificant differences between Cd and HS + La treatment determined by Student’s *t*-test. Cd, control (1 mg·kg^−1^ Cd); HS + La, 1 mg·kg^−1^ Cd + 1 mg·kg^−1^ La at heading stage.

The relative expression of *TaZIP7* was considerably downregulated by La application at the heading stage compared to that of the Cd treatment, whereas the expression of *TaLCT1* was remarkably upregulated. By contrast, the relative expression of *TaHMA2* was unaffected by La addition (Figure 8).

## 4. Discussion

La is not regarded as an essential element for plants, but its beneficial effects on regulating plant growth under Cd stress have been frequently mentioned in previous studies [16,23,42]. For example, Liu et al. [43] revealed that La promotes root growth in Bahiagrass (*Paspalum notatum*) by enhancing root activity, photosynthesis, and respiration. La regulates rice growth by accelerating the absorption of mineral elements [44,45]. La can also promote plant resistance to Cd toxicity by regulating the metabolism of ascorbate and glutathione [24], upregulating the expression of antioxidant enzyme-related genes [46], improving photosynthetic capacity, reducing membrane permeability and malondialdehyde content, and maintaining the activities of catalase and peroxidase [47]. Yang et al. [23] previously revealed that La supplementation at the shooting stage of wheat considerably increases the grain yield under Cd stress. In this study, we consistently found that La promoted the thousand-grain weight of wheat under Cd exposure (Figure 2d). Yang, et al. [23] concluded that the promotion of the grain yield of wheat under Cd exposure by La can be attributed to promotions in photosynthesis, uptake of nutrient elements, and antioxidant ability. In the present work, La supplementation at the heading stage had a stronger influence on the promotion of thousand-grain weight than La application at the shooting stage (Figure 2d). The grain-filling stage from anthesis to maturity is vital for the formation of grain yield [37]. Photosynthesis is important for the generation of energy and the synthesis of proteins, starch, and cellulose in grains [48]. La has been proven to increase the photosynthetic rate by substituting the central Mg^2+^ of chlorophyll [49]. Possibly, the increase in thousand-grain weight by La supplementation at the heading stage is due to the La-promoted photosynthetic rate during the grain-filling stage, which accelerates the generation of energy and synthesis of proteins, starch, and cellulose in grains.

La^3+^ and Cd^2+^ have similar physiological properties because of their nearly similar ionic radii (La: 1.06 Å, Cd: 0.97 Å) [50,51]. Early studies have frequently reported that La inhibits Cd accumulation in plants [22,23,52]. Yang et al. [23] found that La application at the shooting stage reduces Cd concentrations in the roots, stems, leaves, and grains of wheat. In the present work, La addition likewise decreased Cd accumulation in all tissues of wheat, with the exception of the increased Cd concentration in N1 (Figure 3). Previous research has shown that La reduces Cd accumulation in wheat grains by suppressing the absorption and translocation of Cd [23]. One of the reasons for the reduced Cd concentration in wheat grains could be related to the inhibition of Cd absorption and root-to-shoot transport by La application. Another reason could be the upward translocation of Cd from the shoots to the grains through several nodes (junctions between leaves and stems), which have distinct functions in limiting the allocation of Cd to the grains of crops [25,53,54]. The nodes have three types of vascular bundles: EVBs, transit vascular bundles, and DVBs. The bottom nodes (near the roots) have been reported to be essential tissues for limiting the upward transport of Cd to the panicle [55]. In the uppermost nodes, most of the Cd is accumulated in EVBs and DVBs, thereby limiting the transport of Cd to the internodes [53]. N1 has been found to be a key barrier to xylem-to-phloem transport and has the highest Cd retention among all plant nodes [25,54]. The present study revealed that La supply inhibited the transport of Cd from Ni to INi but increased the transfer of Cd from IN_i+1_ to N_i_ (Figure 7). We also found that La supplementation increased Cd accumulation in N1 (Figure 3d and Figure 5) and considerably promoted the percentage of Cd in N1 (Figure 6). Thus, the decreased concentrations in wheat grains could also be attributed to the La-restricted xylem-to-phloem delivery of Cd to the grains by the storage of Cd in the nodes. Numerous studies confirmed that Cd remobilization also contributes a large proportion to the total Cd in grains [34,36,56]. However, in the present work, La application increased the TF_Li-Ni_ values of Cd, and La supplementation at the heading stage had a stronger influence on the TF_Li-Ni_ values of Cd than La supplementation at the shooting stage (Figure 7). We can thus conclude that the La-inhibited Cd accumulation in the wheat grains was not due to the reactivation of Cd from the leaves via the phloem.

The grain-filling stage has been confirmed to be an essential period for final Cd accumulation in crop grains [38]. In the present work, La supplementation at the heading stage had a stronger influence on the reduction in Cd concentrations in the wheat grains (Figure 3a) and the promotion of Cd in N1 (Figure 3b) than La application at the shooting stage. A previous study demonstrated that final Cd accumulation mainly originates from transport redirection through nodes and reactivation from leaves through the phloem to grains during grain filling [25]. *ZIP7*, *LCT1*, and *HMA2* have been confirmed to be implicated in facilitating the redirection and remobilization of Cd from nodes to rice grains [33,36,57]. The results of the present work revealed that La considerably downregulated the expression level of *TaZIP7* (Figure 8a). Therefore, a possible reason for the remarkable influence on the reduction in Cd concentrations in the wheat grains and the promotion of Cd in N1 by La application at the heading stage was that La suppressed redirection through the nodes and increased the storage of Cd in N1 by regulating the expression of *TaZIP7* during grain filling, thereby hindering Cd delivery to the upper tissues [33]. This explanation is supported by the lowest TF_Ni-INi_ and the highest TF_INi+1-Ni_ in the plants supplied with La at the heading stage among the different treatments (Figure 7). Additionally, *LCT1*, a plasma membrane-localized transporter, is involved in the phloem transport of Cd [36]. Knockout of *OsLCT1* leads to a 50% reduction in Cd in rice grains [36]. The results in this work showed that *TaLCT1* was considerably upregulated by La application (Figure 8b). Previous reports showed that TaLCT1 is implicated in the transport of Ca, Na, Rb, and K [58,59]. The underlying reason for this phenomenon is that plants actively redistribute a large number of nutrient elements to the grains by inducing the upregulation of *TaLCT1* for grain formation during grain filling [60]. The explanation is supported by the increased thousand-grain weight (Figure 2) in the current study. Moreover, the upregulation of *TaLCT1* corresponded to increased TF_Li-Ni_ by La supply, and La supplementation at the heading stage had a stronger influence on TF_Li-Ni_ than La supplementation at the shooting stage (Figure 7). TaHMA2 is localized in EVBs and DVBs and involved in Cd reloading from the intervening parenchyma tissues to the phloem of DVBs in the nodes [61]. La addition in this study did not affect the expression of Ta*HMA2* (Figure 8c), indicating that the La-limited Cd allocation was not due to the regulation of *TaHMA2*.

In conclusion, La application increased thousand-grain weight and suppressed Cd accumulation in the roots, stems, leaves, and grains of wheat. La addition considerably decreased TF_N3-IN3_, TF_N2-IN2_, and TF_N1-IN1_ and increased TF_IN4-N3_, TF_IN3-N2_, and TF_IN2-N1_. Compared with La supplementation at the shooting stage, La supplementation at the heading stage had a stronger influence on the increase in Cd storage in N1 and the reduction in Cd concentrations in the other tissues. La application downregulated the expression of *TaZIP7*. Overall, the effective reduction in Cd in the wheat grains by La application at the heading stage was probably a consequence of the successful reduction in Cd redirection and the promotion of Cd storage in the nodes resulting from the downregulation of the expression of *TaZIP7* during the grain-filling stage.

## Data Availability

Data are contained within the article.

## Supplementary Materials

The following supporting information can be downloaded at: https://www.mdpi.com/article/10.3390/plants13202921/s1, Table S1. The physic-chemical characteristics of the soil used in this experiment; Table S2. Sequences of specific primers used for qRT-PCR.
