# Supplementary material for: Application of Lanthanum at the Heading Stage Effectively Suppresses Cadmium Accumulation in Wheat Grains by Downregulating the Expression of TaZIP7 to Increase Cadmium Retention in Nodes
Source: Plants (Basel). 2024 Oct 18;13(20):2921. doi: 10.3390/plants13202921 (PMC11510972; doi:10.3390/plants13202921)
Supplement: Supplementary file 1 [file plants-13-02921-s001.zip › plants-3225181-supplementary.pdf]

**Supporting Information**

**Table S1** The physic-chemical characteristics of the soil used in this experiment

| Soil texture | pH | OM ($mg \cdot kg^{-1}$) | CEC ($mmol \cdot kg^{-1}$) | TN ($g \cdot kg^{-1}$) | TP ($mg \cdot kg^{-1}$) | TK ($g \cdot kg^{-1}$) | background Cd (($mg \cdot kg^{-1}$) |
|---|---|---|---|---|---|---|---|
| Loam | 6.02 | 21.20 | 8.16 | 0.72 | 586.2 | 0.74 | 0.44 |

Note: OM organic matter, CEC cation exchange capacity; TN, TP, TK indicate total nitrogen, total phosphorus and total potassium, respectively.

**Table S2** Sequences of specific primers used for qRT-PCR

| Gene name | Forward primer(5'-3') | Reverse primer (5'-3') |
|---|---|---|
| TaZIP7 | ACAGGCAGTATGTTSGGACGTAG | CAGCAAGTGATGGCCTATGTCG |
| TaLCT1 | TGTGCTGTTTGGTTGGTCCT | LCT1-R CCGATTGGCGACCTTCGATA |
| TaNRAMP5 | CTCCTCTGGGTGATTCTGATTG | CGGCTTTGGATACTCGGTCT |
| TaHMA2 | GGGCATCCGCTTATTTGG | TTCCACTGCCTTTCTCCCTC |
